# Invasive plants reduce functional feeding diversity and trophic interactions of insect herbivores on a remote tropical island

**DOI:** 10.1371/journal.pone.0349238

**Published:** 2026-06-11

**Authors:** Maia R. Supple, Jaemin Lee

**Affiliations:** 1 Department of Environmental Sciences, Policy, and Management, University of California, Berkeley, California, United States of America; 2 Department of Integrative Biology, University of California, Berkeley, California, United States of America; University of Udine: Universita degli Studi di Udine, ITALY

## Abstract

Invasive plants are a driver of global insect decline, yet their impacts on trophic interactions in insular tropical island ecosystems remain poorly understood. We investigated patterns of insect herbivory in the lowland rainforest of Moʻorea, French Polynesia—a remote tropical island in the South Pacific—on a total of twelve species of native, naturalized (introduced, non-invasive), and invasive host plants common in the forest. Using the functional feeding group–damage type system—a functional approach commonly applied in paleoecological studies that investigates traces of insect and fungal herbivory—we quantified the diversity and abundance of herbivory traces on fresh leaf litter and constructed weighted bipartite networks to assess community structure. Our results show a significantly reduced trophic interaction on invasive plants, which experienced substantially lower functional diversity and intensity of insect herbivory across all functional groups (e.g., stylophytic feeding, galling, margin feeding) compared to native and naturalized ones. Network analysis confirms that invasive plants held peripheral, weakly connected positions, whereas native and naturalized plants had central roles in integrating the trophic interactions. The high connectivity of naturalized plants in the network analysis, as well as comparable herbivory diversity and intensity to native species, suggest their successful assimilation into the local community, implying that susceptibility to herbivory may suppress the population expansion and invasion of some introduced plants. The prevalence of rare and specialized interactions and the power-law distribution of the number of host plants each damage type occurs on, observed in our study, mirrored the global patterns of host plant usage by insect herbivores—dominated by rare and specialized interactions—further suggesting the functional feeding group–damage type system as a robust and efficient tool for comparative insect herbivory investigations.

## Introduction

Herbivorous insects account for roughly half of the exceptional insect biodiversity [1], and diversification rates in many clades are strongly correlated to herbivory [2]. In particular, many tropical insect herbivores have narrow dietary breadths, feeding on a single host species or a small number of closely related plants in the same family [3]. Consequently, anthropogenic introduction of nonnative plants [4]—and the subsequent establishment of self-sustaining reproductive populations (naturalization) and rapid range expansion (invasion) [5]—would pose a severe threat to tropical insect communities. These invasive plants are now a major contributor to the global decline in insect populations [6,7], triggering cascading effects across the food web and ecosystem functions [8,9].

The dynamics between insect herbivores and novel, introduced plants are shaped by multitudes of factors and can vary by context. For instance, plant invasions often negatively impact specialist herbivores and pollinators, whereas they can increase the abundance of generalists [7]. Reciprocally, herbivory rates can also affect the success of plant invasion. Introduced plants may benefit from the absence of pre-adapted specialists (enemy release hypothesis [ERH]) [10,11], or they may be attacked more by generalist herbivores to which they are defensively naïve (biotic resistance hypothesis [BRH]) [12]. Currently, mixed support exists for both hypotheses, and herbivore responses to introduced plants often depend on the phylogenetic and allelochemical similarities between the introduced and native plants [13].

While the tropics facilitate the greatest plant and insect biodiversity, rates of insect herbivory, degrees of host specialization, and plant defenses [3,13,14], research remains heavily biased towards subtropical to temperate regions of the northern hemisphere [15]. This geographic bias is compounded by a taxonomic one, where research and conservation efforts disproportionately focus on pollinators and charismatic beetles [16], despite the critical ecological roles of folivorous insects. This knowledge gap is most prominent in remote tropical oceanic islands. These insular ecosystems, characterized by high endemism and low overall species richness, are particularly vulnerable to biological invasions [17]. While historical hypotheses have postulated that plants on islands experience reduced herbivory, recent data indicates that invertebrate herbivory on island ecosystems is comparable to that of the mainlands [18]. Therefore, research efforts towards a fundamental understanding of plant–insect interactions on tropical oceanic islands, under its ever-changing landscapes of plant invasions, are warranted.

We investigated the patterns of herbivory across native, naturalized (introduced, established but noninvasive), and invasive plants in Moʻorea, French Polynesia—a remote, tropical volcanic island in the South Pacific Ocean whose native flora is heavily threatened by biological invasions. We quantified functional feeding diversity and herbivory intensity by analyzing traces of foliar herbivory on leaf litter by applying the functional feeding group–damage type system—commonly applied in paleoecological research [19]. We hypothesized that invasive plants would: 1) receive less diverse and intense insect herbivory; 2) experience less complex and specialized interactions compared to native plants; and 3) display distinct herbivory patterns from those of naturalized plants.

### Functional feeding group–damage type system

Insect and fungal herbivory damages on leaves are distinctively recognized by a thickened, upraised reaction rim along the feeding margin (S1 Fig) [20]. We identified these traces as **damage types** (DTs), which are functional units of insect and pathogen herbivory traces defined by distinct morphological features such as size, shape, position, specific reaction tissues, and micromorphology [19,21].

Foliar DTs are categorized into nine **functional feeding groups** (FFGs)—eight insect-derived groups (hole feeding, margin feeding, skeletonization, surface feeding, piercing & sucking, oviposition, mining, galling) and one pathogen group (fungal)—which are categorized based on similar feeding styles [19]. These FFGs reflect specific dietary guilds of insect herbivores (e.g., leaf chewers, leaf suckers, gallers, leaf miners), and are organized into four broader **feeding classes** based on how insect mouthparts or ovipositors primarily interact with live plant tissues: ectophytic (external chewing), stylophytic (piercing), endophytic (internal feeding), and pathogen [19,21]. This system reflects varying degrees of morphological and behavioral specialization in insect and fungal herbivores. For example, ectophytic chewing is often more generalistic, whereas stylophytic feeding of leaf tissues often requires behavioral coordination to empty specific mesophyll cells, and endophytic feeding (galling and mining) demands precise oviposition and tissue-specific larval adaptation [22–24].

We selected the FFG–DT system to investigate insect herbivory dynamics for several reasons. While standard neoecological studies typically focus on direct insect collection or observation, these approaches can be time- and labor-intensive. First, taxonomic identification of insects often relies on specialized taxonomic expertise, particularly for immature stages including miners and gallers, and abundant, undescribed rare species in the tropics add additional challenges to insect identifications in remote tropical islands [25]. Additionally, direct sampling is often biased toward more easily accessible plants (herbs, shrubs and the lower stratum of trees) and slow-moving insects, frequently missing agile herbivores or canopy-dwelling species [25–27]. In tropical forests where insect herbivore communities and plant–herbivore interactions are characterized by a large number of rare species and interactions [25,28], such sampling methods are likely to omit many rare interactions.

The FFG–DT system—largely developed and applied in paleoecological research [19,21,29]—instead focuses on the functional units of insect feeding traces. Mature leaves cumulatively preserve traces of herbivory across the leaves’ lifespan and the tree’s vertical structure, which readily provide insights into rare interactions that “snapshot” sampling of insects can omit. Furthermore, because functional feeding diversity correlates positively with taxonomic richness of the damage makers [30], this method provides a time- and cost-effective, robust proxy for comparative herbivore diversity, which makes the FFG–DT system uniquely suited for comparing herbivore communities across time, space, and host lineages [30,31].

## Methods

### Study area and organisms

Moʻorea is a small tropical island located in the Windward group of the Society Islands, French Polynesia. Situated in the central South Pacific Ocean (~17°S), it lies approximately 17 km northwest of the younger and larger island of Tahiti, but remains isolated from major continental landmasses by over 4,000 km. Geologically, Moʻorea was formed 2.0–1.5 million years ago during the Society archipelago hotspot event [32,33], and covers an area of ca. 135 km^2^. The island’s topography is defined by 57 main valleys separated by high volcanic peaks, the tallest of which, Mont Toheia, reaches 1,207 m. Climatically, Moʻorea sits along the southeastern edge of the South Pacific Convergence Zone (SPCZ). It experiences a wet season from November to April, driven by both SPCZ and orographic effects, and a dry season from May to October, when rainfall is primarily orographic [34].

The tropical flora of Moʻorea spans an elevational gradient, from coastal mesic to dry forests, lowland rainforests in valleys and slopes, shrublands on ridges and peaks, and montane cloud forests in the high elevations [35]. This landscape is also defined by a long history of human influence. The arrival of the Mā’ohi (indigenous Tahitians) around 900–1000 AD was associated with extensive forest clearing, the loss of littoral and primary forest constituents, and the introduction of canoe plants [36–38]. Coastal lowlands and valleys were densely settled by Mā’ohi until the indigenous populations collapsed after European arrival in the 18th century [39]. Many Polynesian-introduced plants then became integral to regenerating secondary forests in these areas [35,40]. A second sharp floristic turnover occurred shortly after ca. 1770, when the first Europeans visited the island [38]. Upon French colonization of Tahiti ca. 1840, many nonnative plants were introduced for agricultural and horticultural uses [41]. This culminated in the 1930s with the introduction of aggressive invaders like *Spathodea campanulata* (African tulip tree) and *Miconia calvescens* (purple velvet tree), which have since become major ecological threats to the biota of both Tahiti and neighboring Moʻorea [41]. Today, invasive species exceed native flora in both species richness and land coverage, restricting native vegetation to just ~6% of the island, primarily restricted to high-elevation montane cloud forests [35,42].

**Fig 1:**
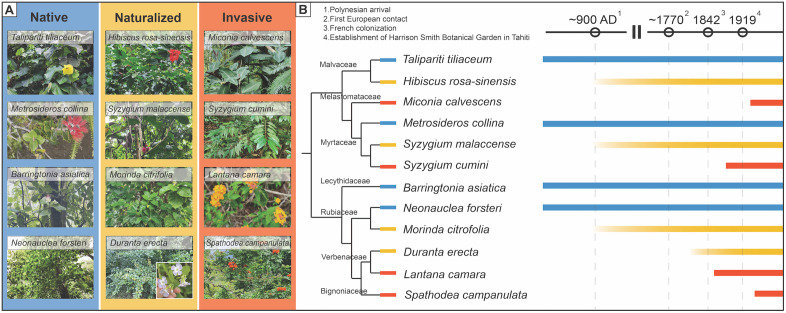
The twelve study plants selected from the tropical lowland rainforests of the ʻŌpūnohu Valley, Moʻorea, French Polynesia. **(A)** The twelve study plants (four per native, naturalized, and invasive categories), illustrating diverse growth forms ranging from shrubs to canopy trees. **(B)** Phylogenetic relationships and estimated arrival times of each species on Moʻorea/Tahiti, French Polynesia.

Fieldwork was conducted in the lowland rainforests of the **ʻ**Ōpūnohu Valley during October–November 2023. We selected twelve abundant woody eudicot species, divided into three categories in equal numbers (Fig 1). **Native** plants, indigenous to the island, include *Talipariti (Hibiscus) tiliaceum*, *Metrosideros collina*, *Barringtonia asiatica*, and *Neonauclea forsteri* [43–45]. While *T. tiliaceum* and *B. asiatica* are sometimes considered canoe plants in some parts of Polynesia, charcoal remains from the **ʻ**Ōpūnohu Valley suggest that they were indigenous to Moʻorea [37]. **Invasive** plants include *Miconia calvescens*, *Syzygium cumini*, *Lantana camara*, and *Spathodea campanulata* [41]. Lastly, **naturalized** plants—introduced by humans that are established but not rapidly expanding in their range—include three species of Mā’ohi introduction (*Hibiscus rosa-sinensis*, *Syzygium malaccense*, and *Morinda citrifolia*) and one European introduction (*Duranta erecta*). Although *D. erecta* is invasive in other Pacific islands, the rate of its expansion is not considered invasive in French Polynesia (J.-Y. Meyer, pers. comm.). The native, naturalized, and invasive plant groups are collectively referred to as “host plant categories” hereinafter.

Several plant traits influence insect herbivory, including the systematic affinities, allelochemistry, leaf nutritional quality, morphology and apparency (e.g., leaf sizes, plant growth forms), and ecological strategies (e.g., successional status, competitor–stress tolerator–ruderal [CSR] strategies) [25,46,47]. These traits vary across our twelve study plants; however, qualitative assessment suggests that such variation is not skewed towards a specific plant category. For instance, we controlled for leaf nutritional quality by excluding nitrogen-fixing plants, which are known to receive greater herbivory in the tropics [48]; this excluded some legume species that are common in the ʻŌpūnohu rainforest (e.g., *Inocarpus fagifer*, *Falcataria falcata*). Globally, rosid eudicots on average experience nearly twice the herbivory rate of the asterids [47], and while this overall global pattern may not accurately predict local community patterns, we balanced our twelve study plants to include six species from both clades, each containing two species of the three host plant categories. Similarly, while the allelochemistry of the study species was not quantified, qualitative assessment suggests that major secondary compounds (e.g., polyphenols, terpenoids, glycosides) are present across all host plant categories. Other morphological and ecological traits were similarly variable but showed no systematic skew toward any specific group (S1 File).

Study plants were identified and selected along a 3 km transect (200–400 m elevation) following the road from Lycée Agricole d’Opunohu to the Belvédère, and le Col des Trois Cocotiers trail in the ʻŌpūnohu Valley. We identified five individual trees per species, located 5–10 meters from the road/trail. Conspecific trees were separated by at least 10 m; the mean conspecific distance was 238 m (s.d. = 368 m, max = 1,734 m; S1 File), reflecting the heterogeneous forest structure. For each of the 60 study plants, we collected 50 freshly fallen, undecomposed leaves from the forest floor within a 3 m radius of the tree trunk. Sampling proceeded outward from the tree trunk to ensure randomization. The 3,000 collected leaves were labelled and brought back to the Richard B. Gump South Pacific Research Station for detailed examination.

### Herbivory diversity and intensity

We classified herbivory damages (examples shown in S1 Fig) using the 150 established DTs of Labandeira et al. (2007) [19]. Additionally, we identified seven DTs that did not fit into the existing classifications, which were described and recorded as new morphotypes (MDTs; S2 File).

We analyzed herbivory patterns across the host plant categories by comparing the DT diversity and herbivory intensity. We constructed diversity accumulation curves by leaf specimens to understand the cumulative DT richness across the host plant categories, using the vegan package [49]. Additionally, we calculated the mean DT richness across the host plant categories by averaging the cumulative DT richness of each tree (N = 20 per category).

Herbivory intensity was quantified by measuring damage type frequency and percent area damage (PAD) [21]. Damage type frequency is defined as the proportions of damaged leaves out of the 50 sampled leaf litters per tree and is treated as binary for each leaf (present/absent). PAD was quantified using a 2 x 2 mm grid censusing method, chosen for its feasibility over digital tracing in field conditions. We counted grid overlaps for each DT and cumulatively added them to calculate PAD of all DTs per leaf. Fungal damages were excluded from calculating this metric, as their extensive exophytic surface growth can overlap with insect damages and distort area estimates.

For statistical analyses, the three herbivory metrics (DT richness, frequency, and PAD) were compared. PAD for each tree was log-transformed as log(PAD + 0.001) to reduce skewness and accommodate zeros. To account for the hierarchical structure of the data (leaves nested within trees, trees nested within species), we employed Linear Mixed-Effects Models (LMMs)—and for binary frequency data, General Linear Mixed Models (GLMMs) with a binomial error distribution—using the lme4 and ImerTest packages in R [50]. PAD was log-transformed to meet assumptions of normality. We treated the host plant category as a fixed effect, while plant species was included as a random effect. Model fit and residuals were verified using the DHARMa package [51]. The significance of fixed effects was evaluated using Type III Analysis of Variance (ANOVA) with Satterhwaite’s approximation for degrees of freedom. Post-hoc pairwise comparisons were conducted using estimated marginal means via the emmeans package [52] with Tukey’s HSD adjustment. All analyses were performed in R version 2024.09.1 + 394 (R Core Team, 2024).

### Patterns by functional feeding groups and feeding classes

Because functional feeding groups and feeding classes reflect varying degrees of morphological and/or behavioral specialization [19,53], we compared DT richness and frequency across nine foliar FFGs and four feeding classes (ectophytic, stylophytic, endophytic, and pathogen) across the host plant categories. We followed the same statistical tests for the DT richness and frequency as above-described; host plant category and FFG/feeding class were treated as fixed effects, while plant species were included as random effects.

### Bipartite network analysis of host plant–damage type

To explore the complexity and specificity of plant–DT interactions, we constructed weighted bipartite networks, linking plant species (lower level) with DTs recorded on their leaves (upper levels) [54]. Interaction strength was defined as the number of observations for each plant–DT link. To reduce noise from rare, insubstantial interactions, we limited the analysis to DTs with at least three occurrences. We then quantified the structural properties of the network using the bipartite package [55]. Specifically, we estimated weighted nestedness using the weighted NODF index, network-wide interaction specialization using H_2_’, and co-occurrence patterns using C-score (S1 Table).

To characterize the functional role of individual taxa, we extracted species-level metrics for each plant and DT, including species strength, degree, proportional similarity index (PSI), weighted closeness, proportional similarity, resource range, species specificity index, proportional generality, and partner diversity (S1 Table). These metrics capture various interaction characteristics, including node importance, specificity, and centrality, relative to other nodes. To evaluate how plant introduction history shapes host use, plant-level metrics were coded by host plant categories; conversely, DT-level metrics were coded by feeding classes to illustrate specialization and centrality by feeding mode. Lastly, we performed Principal Component Analysis (PCA) on the scaled species-level metrics. Plant-level PCA was color-coded by host plant category and DT-level PCA by feeding classes, with 95% confidence ellipses. DT-level PCA was restricted to feeding groups represented by at least two DTs. The top seven contributing variables were extracted to identify the primary drivers of multivariate structure. The analysis and visualization were done using packages dplyr, tidyr, bipartite, FactoMineR, factoextra, and ggplot2 [55–60].

## Results

### Herbivory diversity and intensity across native, naturalized, and invasive plants

Across the 3,000 leaf litter samples from the ʻŌpūnohu rainforest, we identified a total of 79 DTs. Thirty DTs were found on a single host plant (38%), which were largely stylo- and endophytic types. Twenty-four DTs occurred on two to three distantly related host species (30.4%), and eleven DTs were found across four to eleven distantly related plants (13.9%). Overall, the occurrence distribution followed a concave power law (S2 Fig). The remaining 14 DTs (17.7%) were found on all twelve study species and were predominantly represented by ectophytic DTs.

**Fig 2:**
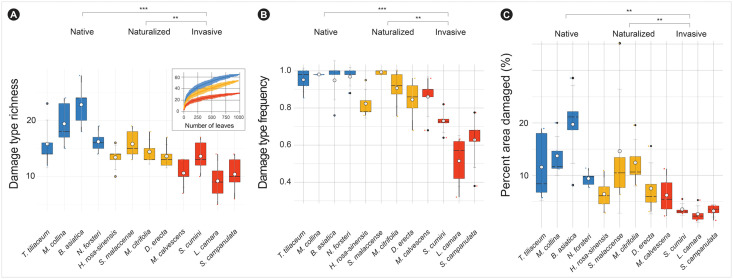
Patterns of herbivory functional diversity and intensity on native, naturalized, and invasive host plants in the ʻŌpūnohu rainforest, Moʻorea, French Polynesia. **(A)** Damage type (DT) richness. The boxplots show values by species, and the small box in the upper-right corner shows DT diversity accumulation curves by leaf specimens across the host plant categories (shaded area showing 95% confidence intervals). **(B)** DT frequency, representing the proportions of damaged leaves based on binary presence/absence assessment. **(C)** Percent area damaged, representing the proportions of the leaf tissues removed by herbivory. Herbivory metrics were compared at the host plant category level, but individual species values are displayed herein to show the variation across species within each category. Herbivory metrics of each species were calculated by averaging the values for each of the five trees per species, which in turn derive from the fifty leaf litter samples per tree. Plot details: white circle = mean, mid-bar = median, bracketed vertical bar = standard deviation, box = upper and lower quartiles, whiskers = maximum and minimum, dots = value for individual trees. Statistical notations: Asterisks indicate statistical significance of pairwise comparisons between host plant categories, based on linear mixed-effects models (A and C) and generalized linear mixed models (B) (*p < 0.05, **p < 0.01, ***p < 0.001).

Invasive plants consistently demonstrated significantly lower herbivory metrics than native and naturalized species. Invasive plants showed reduced DT richness (33 cumulative DTs) compared to native (77) and naturalized species (55) (Fig 2A). Invasive plants recorded less frequent DTs (68.2%) compared to native (96.2%) and naturalized (89.1%) species (Fig 2B), and PAD of the invasives (3.89%) was also significantly lower than that of native (13.43%) and naturalized plants (8.69%; Fig 2C). Herbivory metrics for individual species are summarized in S2 Table, and statistical summaries are available in S3 Table.

### Patterns by functional feeding groups and feeding classes

**Fig 3:**
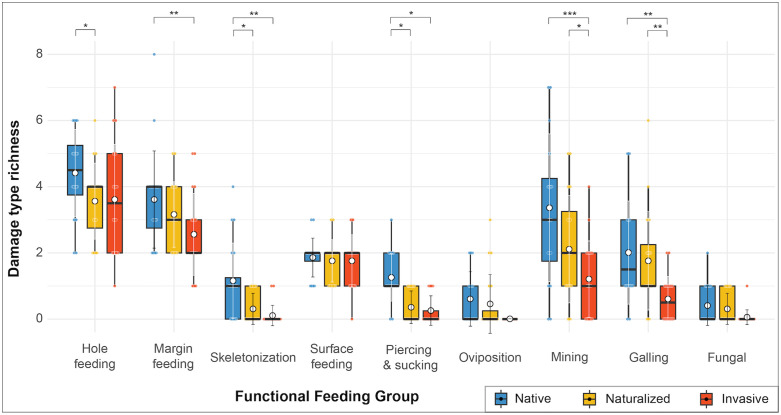
Damage type richness by functional feeding groups across native, naturalized, and invasive study plants in the ʻŌpūnohu rainforest. Plot details: white circle = mean, mid-bar = median, bracketed vertical bar = standard deviation, box = upper and lower quartiles, whiskers = maximum and minimum, dots = value for individual trees. Statistical notations: Asterisks indicate statistical significance of pairwise comparisons between host plant categories, based on linear mixed-effects models (*p < 0.05, **p < 0.01, ***p < 0.001).

Analysis at the FFG level further suggests that native plants support the highest herbivory levels, whereas invasive plants supported the lowest. Invasive plants documented significantly lower mining and galling DT richness compared to both native and naturalized species, as well as less diverse margin feeding, skeletonization, and piercing & sucking DTs compared to native plants. Naturalized plants also received lower DT richness in hole feeding, skeletonization, and piercing & sucking compared to the native species (Fig 3). Invasive plants recorded substantially less frequent margin feeding, piercing and sucking, mining, galling, and fungal DTs compared to both native and naturalized species, and less frequent hole feeding, skeletonization, and surface feeding DTs compared to native plants. Native plants also received significantly more frequent DTs of all insect FFGs except mining compared to naturalized species, but naturalized plants were more frequently damaged by fungal DTs than their native counterparts (S3 File).

At the feeding class level, invasive plants received less diverse ectophytic, styolophytic, and endophytic DTs compared to native plants, and naturalized plants documented less diverse ectophytic DTs than native species (S3 File). Invasive plants received less frequent stylophytic, endophytic, and pathogen damages compared to native and naturalized species, and less frequent ectophytic DTs than native plants. Naturalized plants had less frequent ectophytic and stylophytic DTs, but more frequent pathogen DTs compared to native species (S3 File).

### Bipartite network analysis and specificity of the interactions

**Fig 4:**
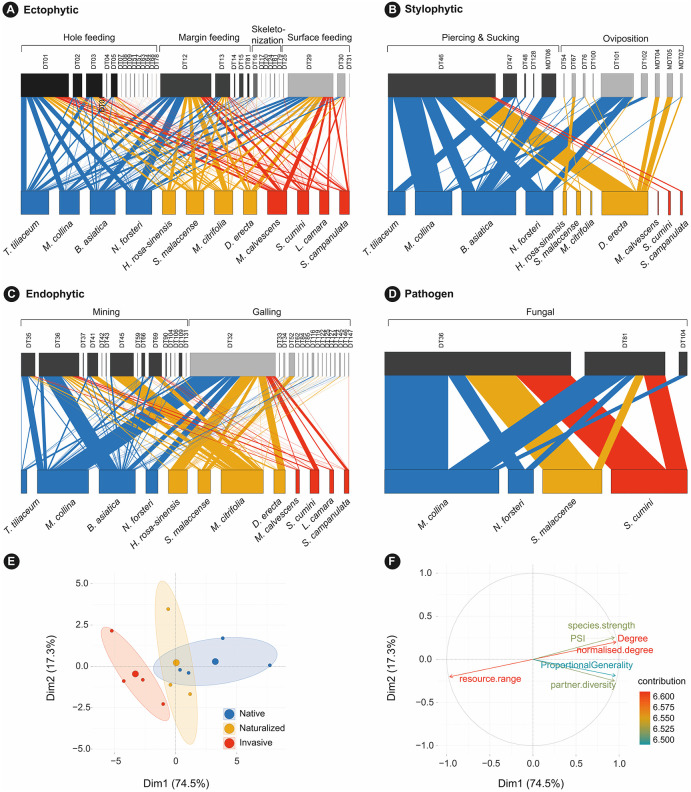
Bipartite network analysis of host plant–damage type interactions. **(A)** Ectophytic damages (hole feeding, margin feeding, skeletonization, and surface feeding). **(B)** Stylophytic damages (piercing & sucking and oviposition). **(C)** Endophytic damage (mining and galling). **(D)** Pathogen damage (fungal). **(E)** Principal Component Analysis (PCA) of host plants, grouped by host plant categories. **(F)** Contribution of the network metrics to the first two PCA axes for host plants.

While bipartite plant–DT interactions are displayed by feeding classes (Fig 4A–4D), all network metrics were calculated using the full network. Weighted by DT occurrence (presence/absence per leaf), the plant–DT network exhibited moderate nestedness (weighted NODF = 34.5) and low specialization (H_2_’ = 3.117). These values indicate largely overlapping plant–DT interactions, where dominant DTs occur across multiple host species. Additionally, the network displayed a moderate checkerboard pattern (C-score = 0.357), reflecting that while overlap is high, there is still partial segregation of DTs among host plants.

In our Principal Component Analysis of the plant-level metrics (Fig 4E and 4F), Dimension 1 (Dim1) accounts for 74.5% of the variation and captures the dominant gradient in host network position. Driven by degree, proportional generality, proportional similarity, partner diversity, and normalized degree, this axis reflects interaction breadth and generalization. Native host plants cluster at the central to high end of Dim1, indicating broad and even DT interactions, whereas invasive hosts occupy lower positions associated with a restricted resource range. Naturalized plants occupy a more central position. In contrast, Dim2 (17.3%) captures weaker variation in resource range, partner diversity, and species strength. This axis reflects how strongly the hosts are integrated into the network rather than their overall degree of generalization. Compared to native plants, invasive and naturalized species are more dispersed across Dim2, suggesting greater variability in their connectivity and network integration.

At the damage type level (S3 Fig), Dim1 captures 73.5% of the variation in network position. This axis is driven primarily by resource range, partner diversity, proportional similarity, normalized degree, and weighted closeness—metrics reflecting interaction breadth. Feeding classes separate clearly along this axis: ectophytic DTs cluster towards broad interaction breadth, whereas stylophytic, endophytic, and pathogen DTs align with restricted and specialized positions. This pattern aligns with the bipartite network, showing the highest connectivity for ectophytic DTs (Fig 4A). Dim2 (12.1%) captures weaker variation associated with weighted closeness, reflecting differences in network embeddedness. Pathogen and ectophytic DTs show a wider spread along this axis, indicating a greater range in network centrality for these groups. All calculated network metrics are available on the Dryad repository.

## Discussion

### Reduced functional feeding diversity and trophic interactions of insect herbivory on invasive plants

Our results demonstrate that invasive plants in the ʻŌpūnohu rainforest host significantly lower functional diversity and intensity of insect herbivory compared to native and naturalized species. Invasive plants recorded significantly lower DT richness (Fig 2A). Notably, the diversity accumulation curve by leaf specimen for invasive plants began to plateau within the 1,000 sampled leaves across four species, whereas the curves for native and naturalized plants did not (Fig 2A), suggesting that additional DT diversity may be detected with further leaf sampling of the native and naturalized study plants. Herbivory intensity was also reduced: invasive plants showed substantially lower DT frequency and PAD compared to their native and naturalized counterparts (Fig 2B and 2C).

When analyzed by FFGs and feeding classes, invasive plants consistently hosted the lowest richness and frequency. Invasive plants recorded less diverse ectophytic (margin feeding, skeletonization) and stylophytic DTs (piercing & sucking) compared to native species, and lower richness of endophytic DTs (galling and mining) compared to both native and naturalized plants. Invasive plants received less frequent DTs of all feeding classes (and all FFGs but oviposition, which was entirely absent on invasive species) compared to native species, and less frequent stylophytic (piercing & sucking), endophytic (mining, galling), and pathogen (fungal) DTs than naturalized plants (Fig 3 and S3 File). These trends suggest that invasive hosts are utilized by a functionally depauperate insect herbivore community. This includes both specialized groups (leaf suckers, gallers, miners) and generalist ectophytic leaf chewers, indicating that invasive plants support fewer interactions across the full spectrum of functional groups.

While based on a limited number of our study plants, our assessment of the DT host specificity by host plant categories, using functional breadth categories [53], largely corroborates that invasive plants poorly support herbivory that requires specialization, also seen in the patterns by functional feeding groups and feeding classes (S4 File). Analogous to the monophagous, oligophagous, and polyphagous dietary breadths of insect herbivores, DTs exhibit an array of functional breadths: specialized (occurring on a narrow range of confamilial host plants), intermediate (host plants across closely related families within an order), and generalized (across distantly related species across orders) [53]. Out of 53 DTs that were recorded at least three times, only seven DTs (stylophytic, endophyic, and pathogen) were categorized as specialized, none of which occurred on invasive study species. The remaining 46 DTs were categorized as generalized DTs, which were most diverse and frequent in native plants and least in invasive species. We acknowledge that an expanded host plant sampling would aid in accurate assessment of the host specificity; however, functional breadth analysis of DTs, while limited to our twelve study plants, further supports that invasive plants support less specialized herbivory (see S4 File for more).

Network analysis of host plants and DTs further supports the patterns observed in herbivory diversity and intensity. PCA ordination of host plant network positions (Fig 4E and 4F) shows a clear separation of invasive plants from native and naturalized study species, which reflects their distinct peripheral positions characterized by lower degree, partner diversity, and species strength (importance of species to interaction partners)—indicating fewer and weaker plant–DT interactions. In contrast, native and naturalized plants occupied central positions, contributing disproportionately to the network connectivity by linking multiple DTs across feeding classes. Notably, host plants separated along the resource range axis, with invasive species exhibiting higher values (more generalized feeding) while native and naturalized species clustering at lower values (more specialized interactions). At the DT level, PCA and bipartite network metrics differentiated feeding modes primarily along Dim1. Ectophytic DTs formed the most connected and widely shared interactions, while endophytic DTs displayed a wide but centralized range. Stylophytic and pathogen damages, conversely, were more specialized and structurally constrained (S3 Fig). Ultimately, the network analysis illustrates that invasive plants are not only less connected but also interact with a narrower subset of functional feeding strategies, reinforcing their peripheral role within the plant–DT network.

Phylogenetic relationships among host plants often explain why certain introduced plants are readily consumed by insect herbivores, as relatedness frequently reflects similarities in allelochemistry [13,61,62]. Because most insect herbivores—particularly specialist guilds like gallers and leaf miners—possess narrow dietary breadths, they are phylogenetically constrained to single host plant species or closely related taxa [3,25,27,63]. While generalist herbivores may exploit hosts across broader phylogenetic distances (often at the family level), their host range often broadly tracks phytochemical similarities [13,22,27]. However, this pattern depends on phylogenetic scale: while broad classes of secondary metabolites may be conserved at the family level, the allelochemistry of congeners can show weak phylogenetic signals [64,65], and host shifts may occasionally occur across distantly related plants due to convergence in defensive traits or nutritional quality [27,63].

From the perspective of native plant relatedness, our study plants present a mixed picture. The invasive *L. camara* (Verbenaceae) and *S. campanulata* (Bignoniaceae) lack known native confamilials in the Society Islands [66]. The highly invasive melastome *M. calvescens* has native and naturalized relatives (including *Astronidium* spp. and *Melastoma denticulatum*), as does *S. cumini*, which belongs to a speciose tree genus with several native and introduced confamilials [66]. However, host relatedness alone fails to explain the observed herbivory patterns: specifically, the naturalized *D. erecta* (Verbenaceae) experienced high degrees of herbivory compared to its invasive confamilial *L. camara*, despite belonging to the same family. Possibly, allelochemical or nutritional differences may drive these disparities; however, the specific phytochemistry of these plants in the ʻŌpūnohu rainforest remains poorly understood. Consequently, while our results suggest that invasive plants generally receive lower herbivory at the community level, more extensive taxon sampling and chemical studies of host plants are needed to better understand the mechanisms behind these differences.

The reduced capacity of invasive plants to support insect herbivores has significant implications across trophic levels. Insects perform critical ecosystem functions, including pollination, seed dispersal, organic matter decomposition, and nutrient cycling, thereby maintaining food webs and energy flow [67,68]. Beyond the direct reduction of trophic interactions, the observed reduction in functional feeding diversity implies a parallel decline in taxonomic richness, given the positive correlation between these metrics [30]. Such reduction in insect diversity and feeding activity can have disproportionately negative impacts on insular ecosystems, where biodiversity and trophic complexity are already simplified [69]. Thus, additional research into the multitrophic effects of plant invasion—and the associated, reduced support for insect herbivores—is warranted.

From a plant population perspective, the reduced herbivory likely contributes to invasion success. As major primary consumers, insect herbivores play a pivotal role in regulating plant regeneration, population dynamics, and community diversity [70–72]. Our results support the enemy release hypothesis, stating that invasive plants escape predation from specialist herbivores. This reduced herbivory may disrupt mechanisms that maintain tropical plant diversity and spatial heterogeneity by preventing conspecific dominance (Janzen–Connell hypothesis) [73–75]. Under relaxed density- or distance-dependent herbivory pressures, invasive plants may grow in closer proximity, which facilitates the formation of monostands. Our study plants include some globally notorious invasive species (*M. calvescens*, *S. campanulata*, *L. camara*), whose success in the remote Pacific Islands appears at least partially attributable to the release from predation pressure. However, we acknowledge that our assessment was limited to foliar herbivory, and the pressure from other herbivore guilds, such as propagule feeders, wood borers, and root feeders [67], remains unknown. Comprehensive studies encompassing multi-organ, if not whole-plant, herbivory are needed to fully understand the impact of insect herbivory on plant community dynamics.

### Acclimatization of insect herbivory on naturalized plants

Patterns of herbivory diversity and intensity were strikingly similar between native and naturalized plants. Damage type richness, frequency, and PAD did not vary substantially between the two groups (Fig 2). Patterns by functional feeding groups and feeding classes did not vary substantially, with the exceptions largely confined to ectophytic and stylophytic DTs (Fig 3 and S3 File). Network analysis also supports this assimilation: naturalized plants occupied intermediate positions with centrality and connectivity metrics similar to native species, indicating that they have been effectively integrated into the local herbivory network.

These results align with previous studies showing that insect herbivore richness on introduced plants increases over time, often becoming comparable to native plants within a few centuries. For instance, leaf-chewing lepidopteran larval richness on over 100 introduced woody plants in Central Europe showed no saturation in herbivore accumulation even after centuries [76], and populations of *Triadica sebifera* (native to East Asia) in the US that were introduced ~200 years ago suffer higher herbivory than those introduced ~100 years ago [77]. The rates of adaptation to new hosts are affected by phylogenetic and phytochemical similarity of the host plants, local community composition, and voltinism and dietary breadths of insect herbivores [25,78].

In the ʻŌpūnohu rainforest, the assimilation of naturalized plants into trophic interactions with insect herbivores likely reflects the residence time of many naturalized species. Species such as *H. rosa-sinensis*, *S. malaccense*, and *M. citrifolia* were introduced by the Mā’ohi centuries ago and have since become foundational elements of the secondary forests that regenerated after the 18^th^-century indigenous population collapse [35]. However, residence time does not fully explain the observed patterns. The naturalized neotropical *D. erecta* (Verbenaceae) was introduced much later (post-European contact) for horticultural purposes, likely contemporaneous to the introduction of its invasive confamilial relative *L. camara*. Despite its more recent arrival and the lack of native relatives on the island, *D. erecta* experiences high degrees of herbivory—including two specialized oviposition DTs—comparable to native and naturalized plants of much older introductions. This stands in sharp contrast to *L*. *camara*, which recorded the lowest herbivory levels. The fact that *D. erecta* has not become invasive in French Polynesia (J.-Y. Meyer, pers. comm.) suggests that rapid colonization by local herbivores may provide biotic resistance, limiting the spread of certain introduced plants while others, like *L. camara*, escape predation.

### Insect herbivory on the remote oceanic island parallels continental patterns

Despite the geographic isolation of the remote, small volcanic island of Moʻorea, our results indicate that insect herbivory levels are comparable to those of continental and other insular ecosystems. While our use of fresh leaf litter differs from fossil-analog leaf pack studies, the standardized richness of 40.6 DTs at 300 leaves in the present study aligns closely with modern samples from temperate deciduous forests in the eastern USA (32.5 DTs) and tropical lowland rainforests in Costa Rica (34.7 DTs) [79]. Similarly, the average DT frequency of 85% in these modern sites [79] is comparable to those of the native and naturalized study plants in this study (native and naturalized species: 82–98%). Average PAD (native and naturalized: 8.7–13.4%) also falls within the 5–20% measured and estimated range of PAD by insect herbivory in other regions of the world [47,80–82].

These findings contribute to our understanding of the relationship between island insularity and insect herbivory. While theory predicts reduced interactions on remote islands due to host–herbivore mismatches from separate dispersal events [83], our data aligns with more recent evidence showing that invertebrate herbivory on islands is comparable to mainland levels, without clear patterns based on island size or isolation [18]. For instance, insect herbivory on islands shows no clear relationship with island area, distance from the mainland, or geologic age in southern European archipelagos [84], whereas herbivory was markedly higher on larger islands among the artificial and forested Barro Colorado Islands in Lago Gatún, Panama, with the isolatedness playing little role [85]. Herbivory patterns in Moʻorea demonstrate that a remote and small tropical island can sustain high functional diversity of insect herbivores. In this context, the drastic reduction of herbivory observed on invasive plants becomes even more significant, as it represents a deviation from a robust, continental-level background herbivory.

The detection of 26 rare DTs (occurring on <3 leaves) out of 79 total suggests that the FFG–DT system effectively captures rare plant-insect interactions. This sensitivity corroborates the findings from insect herbivore surveys, where single feeding individuals often account for 40–50% of trophic links [86,87]. Notably, the distribution of the number of host plants that each DT occurs on is concave, with the majority of DTs restricted to one or a few host plants (S2 Fig). This pattern parallels the Pareto power law distribution observed in global insect dietary breadths, where most insect herbivores are dietary specialists and few are generalists [3], further suggesting that the functional FFG–DT system effectively works as a proxy for taxonomic trophic structure. Considering that DT diversity accumulation curves of native and naturalized plants did not plateau, further sampling would likely reveal an even longer tail of rare interactions.

While the FFG–DT system accurately reflects the underlying structure of herbivore communities—including the prevalence of rare, specialized interactions—it does not provide insights into the taxonomic identity of the herbivores responsible. Our findings indicate that invasive plants support significantly lower functional feeding diversity and trophic interactions of insect herbivores in tropical oceanic islands—where invasive species are actively restructuring ecological networks—warrant further research into the specific taxonomic and phylogenetic diversity of herbivores at risk in these systems. Furthermore, as biological invasions and herbivore adaptations continue, the specific patterns of plant–insect interactions will also change over time. Additional research on the dynamics of plant invasions and insect herbivory across time or broader regions would help elucidate the shifting patterns of plant–insect interactions and eco-evolutionary dynamics of these groups under a rapidly changing global biogeography.

## Conclusion

We investigated the functional diversity and intensity of insect herbivory on a total twelve species of native, naturalized, and invasive plants, abundant in the tropical ʻŌpūnohu rainforest of Moʻorea, using the FFG–DT system. Our results demonstrate that invasive plants experience significantly lower herbivory diversity and intensity compared to the native and naturalized species. This reduction spans all functional categories—from specialized groups (stylophytic damages, galling, mining) to generalized leaf-chewing damages—aligning with the enemy release hypothesis. Bipartite network analysis further supports this distinction: while native and naturalized plants held more central roles in integrating multiple feeding modes, invasive plants occupied peripheral network positions characterized by low connectivity and weak integration. In contrast, naturalized plants showed herbivory patterns and network centrality comparable to native hosts, indicating that insect herbivores have successfully acclimatized to these introduced but non-invasive plants. This suggests that susceptibility to local herbivores may contribute to which introduced plants become invasive while others remain naturalized. Additionally, the comparable patterns between the functional plant–DT and the taxonomic plant–herbivore interactions, including the prevalence of rare specialist interactions and the paralleled power-law distribution of functional and taxonomic dietary breadths, support the efficacy of the FFG–DT system in investigating the eco-evolutionary dynamics of plant invasions.

## Supporting information

S1 Fig Examples of insect and fungal damage types (DTs) from the ʻŌpūnohu rainforest, Moʻorea, French Polynesia. (PDF)

S2 Fig Distribution of the number of host plants that each damage type (DT) occurs on. (PDF)

S3 Fig Principal Component Analysis (PCA) of network metrics for damage types. (PDF)

S1 Table Summary of metrics used in the bipartite network analysis, categorized by level (node, node class, and network), with their ecological interpretations. (PDF)

S2 Table Summary of herbivory metrics for the twelve study plants. (PDF)

S3 Table Statistical summary of herbivory metric comparisons across native, naturalized, and invasive plants. (PDF)

S1 File Ecological and morphological characteristics of the study plants and their locations in the ʻŌpūnohu rainforest of Moʻorea, French Polynesia. (PDF)

S2 File Descriptions of novel damage types identified in the ʻŌpūnohu rainforest, Moʻorea, French Polynesia. (PDF)

S3 File Comparisons of damage type (DT) richness and frequency by functional feeding groups and feeding classes across native, naturalized, and invasive plants in the ʻŌpūnohu rainforest of Moʻorea, French Polynesia. (PDF)

S4 File Host specificity (functional breadth) of damage types across native, naturalized, and invasive study plants in the ʻŌpūnohu lowland rainforest of Moʻorea, French Polynesia. (PDF)
