# Correction: Invasive plants reduce functional feeding diversity and trophic interactions of insect herbivores on a remote tropical island

**DOI:** 10.1371/journal.pone.0356934

**Published:** 2026-08-24

**Authors:** The PLOS One Staff

The hyperlink for Dryad in the Data Availability statement is incorrect. The correct hyperlink is: https://doi.org/10.5061/dryad.4b8gthtrz

The publisher apologizes for the error
