# Supplementary material for: Invasive plants reduce functional feeding diversity and trophic interactions of insect herbivores on a remote tropical island
Source: PLoS One. 2026 Jun 11;21(6):e0349238. doi: 10.1371/journal.pone.0349238 (PMC13257969; doi:10.1371/journal.pone.0349238)
Supplement: S1 Fig — (PDF) [file pone.0349238.s001.pdf]

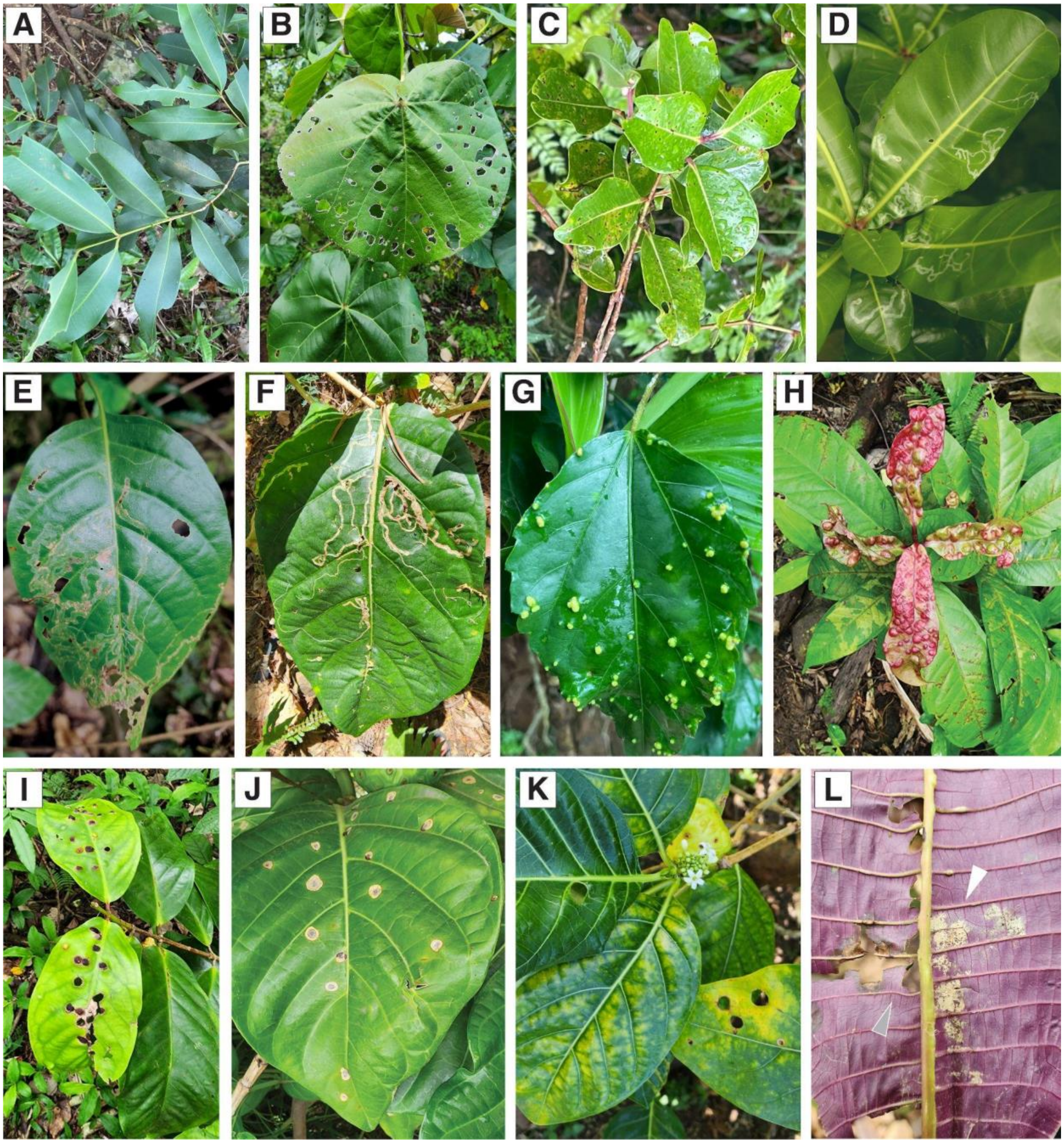

S1 Fig. Examples of insect and fungal damage types (DTs) from the ʻŌpūnohu rainforest, Moʻorea, French Polynesia.

(A) Margin feeding (DT13 & DT14) on *Syzygium cumini*. (B) Hole feeding (DT02) on *Talipariti tiliaceum*. (C) Piercing & sucking (DT46) on *Metrosideros collina*. (D) Mining (DT43) on *Barringtonia asiatica*. (E) Hole feeding (DT02) and mining (DT43) on *Neonauclea forsteri*. (F) Mining (DT41) on *Morinda citrifolia*. (G) Galling (DT32) on *Hibiscus rosa-sinensis*. (H–I) Damages on *Syzygium malaccense*: (H) Galling caused by psyllid nymphs (Hemiptera), which appears as hole feeding after gall is excised (I), identified as DT52. (J–K) Damage on *M. citrifolia*: (J) Circular fungal damage caused noni shot-hole disease, where the excised tissue

resembles hole feeding (identified as DT69). (L) Fungal infection on *Miconia calvescens* caused by the intentionally introduced biological control agent *Collectotrichum gloeosporioides* f. sp. *miconiae*. This damage was identified as mining damage (DT36, white arrow) due to fungal bodies resembling interspersed frass, or as hole-feeding damage (DT05 & DT78; gray arrow) after the necrotic tissue is excised. Panels H–L highlight the complexity of interpreting feeding traces and the potential for morphological mismatch between the final stage and the inferred functional feeding groups.
