# Supplementary material for: Invasive plants reduce functional feeding diversity and trophic interactions of insect herbivores on a remote tropical island
Source: PLoS One. 2026 Jun 11;21(6):e0349238. doi: 10.1371/journal.pone.0349238 (PMC13257969; doi:10.1371/journal.pone.0349238)
Supplement: S2 Fig — (PDF) [file pone.0349238.s002.pdf]

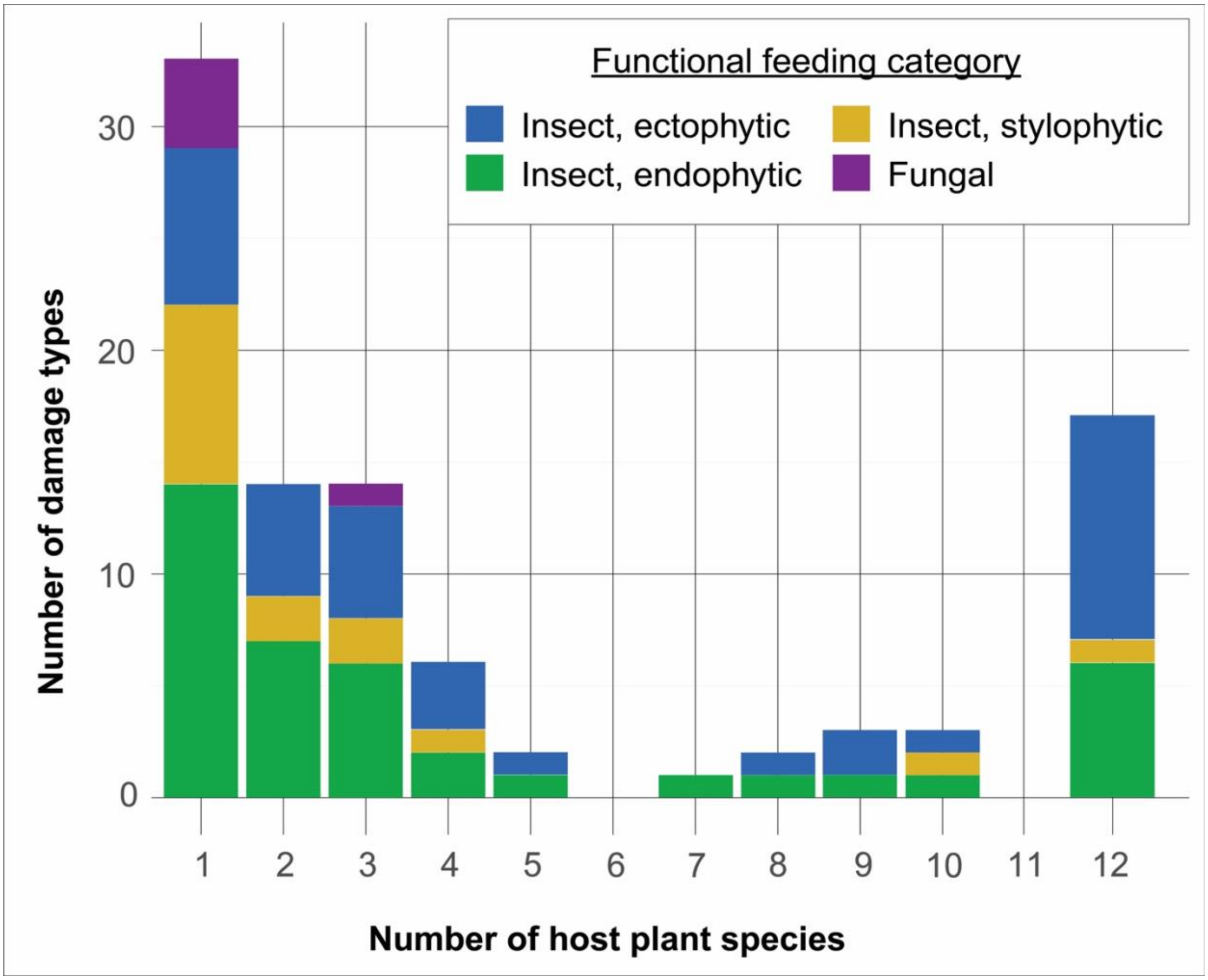

S2 Fig. Distribution of the number of host plants that each damage type (DT) occurs on. The DTs are coded by functional feeding classes (ecto-, stylo-, endophytic, and pathogen). The data show that most DTs occur on a small number of host species, conforming largely to a concave power law distribution similar to global insect herbivore dietary breadths [6]. DTs present on all twelve study taxa typically possessed highly generic morphologies, such as 1-5 mm circular to polylobate holes (DT01–03), circular and shallow margin excision (DT12), and small, circular galls that avoid major veins (DT32).
