# Supplementary material for: Invasive plants reduce functional feeding diversity and trophic interactions of insect herbivores on a remote tropical island
Source: PLoS One. 2026 Jun 11;21(6):e0349238. doi: 10.1371/journal.pone.0349238 (PMC13257969; doi:10.1371/journal.pone.0349238)
Supplement: S3 Fig — (PDF) [file pone.0349238.s003.pdf]

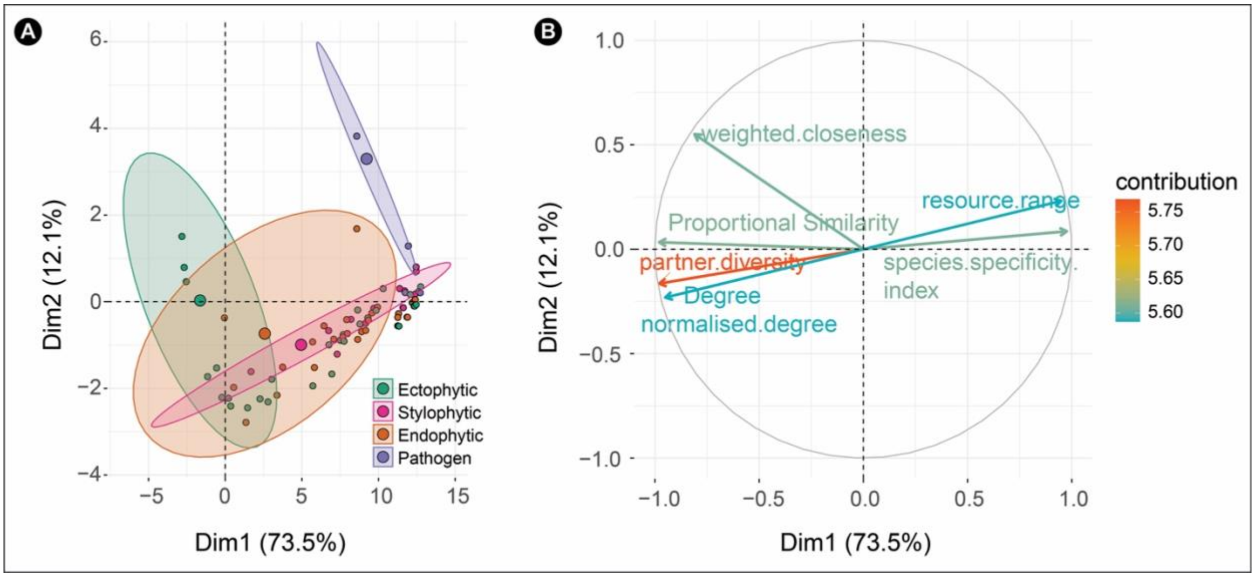


**S3 Fig. Principal Component Analysis (PCA) of network metrics for damage types.**

(A) Damage type PCA grouped by the feeding classes**.** (B) Contribution of network metrics for the first two PCA axes for the DTs.
