# Supplementary material for: Invasive plants reduce functional feeding diversity and trophic interactions of insect herbivores on a remote tropical island
Source: PLoS One. 2026 Jun 11;21(6):e0349238. doi: 10.1371/journal.pone.0349238 (PMC13257969; doi:10.1371/journal.pone.0349238)
Supplement: S1 Table — (PDF) [file pone.0349238.s004.pdf]

S1 Table. Summary of metrics used in the bipartite network analysis, categorized by level (node, node class, and network), with their ecological interpretations. Metrics were calculated using the *bipartite* R package [1], with metric selection for the plant–DT network based on [2].

Metric	Meaning / What It Measures	Type
**Node Level**		
**Species Strength**	How strongly a species (or OTU) influences its partners; sum of dependence values.	centrality
**Degree**	Number of unique partners a species interacts with.	network breadth
**Normalized Degree**	Degree scaled by total possible partners; allows comparison across systems.	standardized breadth
**PSI (Proportional Similarity Index)**	Similarity between actual resource use and resource availability. High PSI = generalist.	specialization
**Weighted Closeness**	How central a node is in the interaction network (shortest paths weighted by interaction strength).	centrality
**Proportional Similarity**	Dissimilarity between expected vs. observed interactions based on abundance.	specialization
**Resource Range**	Breadth of resource types used (e.g., host breadth or environmental space).	specialization
**Node Class Level**		
**Species Specificity Index**	Degree to which a species specializes on particular partners. High = specialist.	specialization
**Proportional Generality**	Effective partners standardized by partner availability; normalized generality.	specialization
**Partner Diversity**	Shannon diversity of partners (accounts for richness + evenness).	specialization / diversity

Entire network level		
**Weighted NODF**	Measures nestedness based on the overlap of interactions	nestedness
**H2’**	Measure of discrimination, calculated in comparison to a network lacking specialization	specialization
**C score**	Mean (normalized) number of checkerboard combinations across nodes	co-occurance

References

1. Dormann CF, Frund J, Bluthgen N, Gruber B. Indices, Graphs and Null Models: Analyzing Bipartite Ecological Networks. TOECOLJ. 2009;2: 7–24. doi:10.2174/1874213000902010007

2. Swain A, Maccracken SA, Fagan WF, Labandeira CC. Understanding the ecology of host plant–insect herbivore interactions in the fossil record through bipartite networks. Paleobiology. 2022;48: 239–260. doi:10.1017/pab.2021.20
