# Supplementary material for: Invasive plants reduce functional feeding diversity and trophic interactions of insect herbivores on a remote tropical island
Source: PLoS One. 2026 Jun 11;21(6):e0349238. doi: 10.1371/journal.pone.0349238 (PMC13257969; doi:10.1371/journal.pone.0349238)
Supplement: S2 Table — (PDF) [file pone.0349238.s005.pdf]

S2 Table. Summary of herbivory metrics for the twelve study plants.
Metrics include damage type (DT) richness, frequency, and percent area damaged. Data were collected from 50 leaves per tree across five individual trees per species. DT richness is presented by both the cumulative total for the species as well as the mean (standard deviation in parentheses) of five individual trees, and DT frequency and percent area damaged values represent the mean (s.d.).

Host plant	DT richness (cumulative)	DT richness (mean)	DT frequency	Percent area damaged
**Native**	70	18.6 (4.41)	0.962 (0.062)	13.4 (4.8)
Talipariti tiliaceum	29	15.8 (4.27)	0.951 (0.063)	11.6 (6.4)
Metrosideros collina	37	19.4 (3.91)	0.980 (0.0003)	13.8 (3.8)
Barringtonia asiatica	45	22.8 (3.90)	0.948 (0.105)	19.7 (7.4)
Neonauclea forsteri	31	16.2 (1.92)	0.968 (0.050)	8.6 (1.7)
**Naturalized**	50	14.2 (2.22)	0.891 (0.102)	8.7 (4.2)
Hibiscus rosa-sinensis	31	13.4 (2.19)	0.822 (0.078)	6.4 (3.1)
Syzygium malaccense	29	15.6 (2.41)	0.992 (0.011)	8.4 (1.6)
Morinda citrifolia	25	14.4 (2.19)	0.906 (0.098)	12.4 (4.5)
Duranta erecta	23	13.6 (2.07)	0.844 (0.110)	7.6 (4.9)
**Invasive**	33	11.0 (3.25)	0.682 (0.171)	3.9 (2.4)
Miconia calvescens	21	10.6 (2.51)	0.860 (0.107)	6.2 (3.7)
Syzygium cumini	23	13.6 (2.88)	0.729 (0.064)	3.5 (1.2)
Lantana camara	19	9.2 (3.49)	0.513 (0.137)	2.6 (1.6)
Spathodea campanulata	19	10.4 (3.21)	0.627 (0.149)	3.2 (1.2)
