# Supplementary material for: Invasive plants reduce functional feeding diversity and trophic interactions of insect herbivores on a remote tropical island
Source: PLoS One. 2026 Jun 11;21(6):e0349238. doi: 10.1371/journal.pone.0349238 (PMC13257969; doi:10.1371/journal.pone.0349238)
Supplement: S3 Table — (PDF) [file pone.0349238.s006.pdf]

**S3 Table. Statistical summary of herbivory metric comparisons across native, naturalized, and invasive plants.**

The table presents comparisons for overall herbivory metrics (DT richness, DT frequency, and PAD). Results include linear mixed model for DT richness and PAD and generalized linear mixed-effects models with a binomial error distribution for DT frequency statistics, and adjusted p-values ($p_{adj}$) for pairwise comparisons between the host plant categories. N: Native, R: Naturalized, I: Invasive.

| Herbivory metrics | Comparisons | P-values ($p_{adj}$) | F-value |
|---|---|---|---|
| DT richness (mean) (Fig 2A) | N–R–I | 0.001** | 14.30 |
| | N–R | 0.299 | - |
| | N–I | < 0.001*** | - |
| | R–I | 0.001** | - |
| DT frequency (Fig 2B) | N–R–I | 0.003** | 10.68 |
| | N–R | 0.223 | - |
| | N–I | < 0.001*** | - |
| | R–I | 0.011** | - |
| Percent area damaged (Fig 2C) | N–R–I | 0.001** | 15.53 |
| | N–R | 0.319 | - |
| | N–I | 0.001** | - |
| | R–I | 0.008** | - |
