# Supplementary material for: Invasive plants reduce functional feeding diversity and trophic interactions of insect herbivores on a remote tropical island
Source: PLoS One. 2026 Jun 11;21(6):e0349238. doi: 10.1371/journal.pone.0349238 (PMC13257969; doi:10.1371/journal.pone.0349238)
Supplement: S1 File — (PDF) [file pone.0349238.s007.pdf]

S1 File. Ecological and morphological characteristics of the study plants and their locations in the ʻŌpūnohu rainforest of Moʻorea, French Polynesia.

Several traits known to influence insect herbivory—including growth form, leaf size, successional status, and Grime's CSR strategies—were analyzed to ensure they did not vary substantially across host plant categories.

<u>Growth form</u>: All taxa are woody eudicots (shrubs to canopy trees). Native taxa are represented by large trees; naturalized species are shrubs to small trees; invasive taxa include both shrubs and large trees.
<u>Leaf Size</u>: Ranges from nanophyll to mesophyll (25–18,225 mm^2). Distribution is balanced across host plant categories, and the invasive M. calvescens represents the largest (the only macrophyll) class.

<u>Successional status</u>: Most taxa are pioneer or early successional species (which typically receive higher herbivory [1]), with the exception of M. collina and N. forsteri that participate in the canopies of late successional forests.

<u>CSR strategies</u>: Most taxa exhibit competitor strategies mixed with stress-tolerant or ruderal traits. This aligns with findings that woody invasives do not strictly conform to the ruderal strategy often seen in herbaceous invasives [2].

In summary, while morphological and ecological traits that may affect insect herbivory vary across the study taxa, there is no systematic bias in the distribution of these traits across the host plant categories.

A summary of morphological and ecological traits of the twelve study taxa.

Species	Origin	Growth form	Leaf size	Successional status	Grime's CSR
Talipariti tiliaceum	Native	Tree, large	Mesophyll	Early successional	C & S & R
Metrosideros collina	Native	Tree, large	Nanophyll	Early to late successional	C & S
Barringtonia asiatica	Native	Tree, large	Mesophyll	Pioneer to early successional	C & S
Neonauclea forsteri	Native	Tree, large	Notophyll	Early to late successional	C
Hibiscus rosa-sinensis	Naturalized	Tree, small	Microphyll	Early successional	C & S
Syzygium malaccense	Naturalized	Tree, small	Mesophyll	Early to mid- successional	C & S
Morinda citrifolia	Naturalized	Tree, small	Mesophyll	Pioneer to early successional	S & R
Duranta erecta	Naturalized	Shrub	Nanophyll	Pioneer	C & S
Miconia calvescens	Invasive	Tree, large	Megaphyll	Early successional	C & R
Syzygium cumini	Invasive	Tree, large	Notophyll	Mid-successional	S & R
Lantana camara	Invasive	Shrub	Microphyll	Pioneer to early successional	C & S & R
Spathodea campanulata	Invasive	Tree, large	Notophyll	Early successional	C & R

Leaf litter samples were collected from a total of 60 trees, five trees each for the twelve study taxa. The trees were 5-10 m away from le Col des Trois Cocotiers trail, in the elevational range of 200-400 m of the tropical lowland rainforest of ‘Ōpūnohu Valley, Mo’orea, French Polynesia (more details in the Methods of the main text).

Species (site)	Latitude	Longitude	Species (site)	Latitude	Longitude
**Native plants**					
Talipariti tiliaceum	-17.541400	-149.827146	*Barringtonia asiatica*	-17.540681	-149.826782
(S2, 7, 27, 53, 59, respectively)	-17.543037	-149.829854	(S4, 39, 52, 54, 48, respectively)	-17.536696	-149.829753
	-17.541405	-149.828675		-17.544869	-149.835951
	-17.541379	-149.827283		-17.544725	-149.835742
	-17.542145	-149.828241		-17.543144	-149.830911
Metrosideros collina	-17.541394	-149.828223	*Neonauclea forsteri*	-17.542697	-149.828906
(S28, 29, 31, 34, 50, respectively)	-17.541349	-149.828936	(S6, 13, 18, 22, 47, respectively)	-17.542134	-149.827337
	-17.541330	-149.828587		-17.541970	-149.828346
	-17.541649	-149.828693		-17.542954	-149.827866
	-17.541262	-149.828961		-17.542551	-149.829339
**Naturalized plants**					
Hibiscus rosa-sinensis	-17.543071	-149.829733	*Morinda citrofolia*	-17.539250	-149.827705
(S8, 9, 14, 32, 49, respectively)	-17.541245	-149.826971	(S36, 37, 41, 46, 58, respectively)	-17.539562	-149.827705
	-17.540757	-149.826789		-17.545159	-149.839651
	-17.540563	-149.826530		-17.546662	-149.8417
	-17.535723	-149.832470		-17.544535	-149.839674
Syzygium malaccense	-17.541691	-149.826976	*Duranta erecta*	-17.537359	-149.829507
(S12, 19, 26, 33, 51, respetively)	-17.542327	-149.828289	(S38, 40, 42, 44, 55, respectively)	-17.543563	-149.830257
	-17.543522	-149.82792		-17.546678	-149.840998
	-17.541818	-149.827466		-17.537332	-149.829402
	-17.543511	-149.82783		-17.535667	-149.832503

**Invasive plants**					
Miconia calvescens	-17.543100	-149.827891	*Lantana camara*	-17.539901	-149.827189
(S5, 10, 24, 57, 60, respectively)	-17.540172	-149.827588	(S11, 3, 35, 43, 45, respectively)	-17.539845	-149.827078
	-17.542067	-149.828507		-17.539611	-149.827379
	-17.542740	-149.827711		-17.538966	-149.827081
	-17.540137	-149.827349		-17.546842	-149.841768
Syzygium cumini	-17.540649	-149.826121	*Spathodea campanulata*	-17.539645	-149.826596
(S15, 17, 20, 23, 30, respectively)	-17.541631	-149.828294		-17.541240	-149.828537
	-17.541786	-149.82793	(S16, 21, 25, 56, 1, respectively)	-17.543661	-149.827854
	-17.541900	-149.827611		-17.539573	-149.826384
	-17.541525	-149.828936		-17.540089	-149.826934

References

1. Mariano NA, Martínez-Garza C, Alcalá RE. Differential herbivory and successional status in five tropical tree species. RevMexBiodiv. 2018;89. doi:10.22201/ib.20078706e.2018.4.2347

2. Montesinos D. Fast invasives fastly become faster: Invasive plants align largely with the fast side of the plant economics spectrum. Journal of Ecology. 2022;110: 1010–1014. doi:10.1111/1365-2745.13616
