# Supplementary material for: Invasive plants reduce functional feeding diversity and trophic interactions of insect herbivores on a remote tropical island
Source: PLoS One. 2026 Jun 11;21(6):e0349238. doi: 10.1371/journal.pone.0349238 (PMC13257969; doi:10.1371/journal.pone.0349238)
Supplement: S2 File — (PDF) [file pone.0349238.s008.pdf]

S2 File. Descriptions of novel damage types identified in the ‘Ōpūnohu rainforest, Mo‘orea, French Polynesia.

The file details seven unique damage types recorded in this study that do not fit the established classification system of Labandeira et al. (2007). These novel types, designated as MDT01–MDT07, encompass one piercing & sucking, three oviposition, and three fungal damage types.

MDT01: fungal (A)
- Polylobate, raised blotches, waxy to the touch. Distribution ranges from small, dispersed spots (<1 mm in diameter) across the leaf to larger patches (2 mm or more) on the adaxial surface. Clusters near 2nd degree veins, avoiding 1st degree veins.
- Host plants: *Neonauclea forsteri*, *Syzygium malaccense*

MDT02: fungal (B)
- Patches of discolored, slightly indented tissues, with clearly defined or diffuse margins. Patch sizes range from <1 mm to 3-4 mm in diameter.
- Host plant: *Neonauclea forsteri*

MDT03: fungal (C)
- Circular white patches (~1 mm) that are rimmed by three to four triangular flaps that are raised and open outward on the adaxial leaf surface.
- Host plant: *Metrosideros collina*

MDT04: Oviposition (D)
- Shallowly conical, exophytically attached structures (<1 mm in diameter) on the abaxial leaf surface that have a centrally darkened and well-defined center. Can be removed from leaf exterior easily. Likely an ovipositional structure of a sternorrhynchan hemipteran.
- Host plant: *Duranta erecta*

MDT05: Oviposition (E)
- Black trails following venation of adaxial and abaxial leaf surface; start thinner and slightly thicken along the vein. Have a defined terminal chamber. Insect molt pictured at the end terminal.
- Host plant: *Duranta erecta*

MDT06: piercing and sucking (F)
- Patches of darkened leaf tissue with small, raised bumps clustered within them. Occurring on both sides of the leaf surface.
- Host plant: *Barringtonia asiatica*

MDT07: Oviposition (G)
Long segmented damage, raised from leaf surface tissue, ~1cm.
Host plants: *Neonauclea forsteri*, *Duranta erecta*

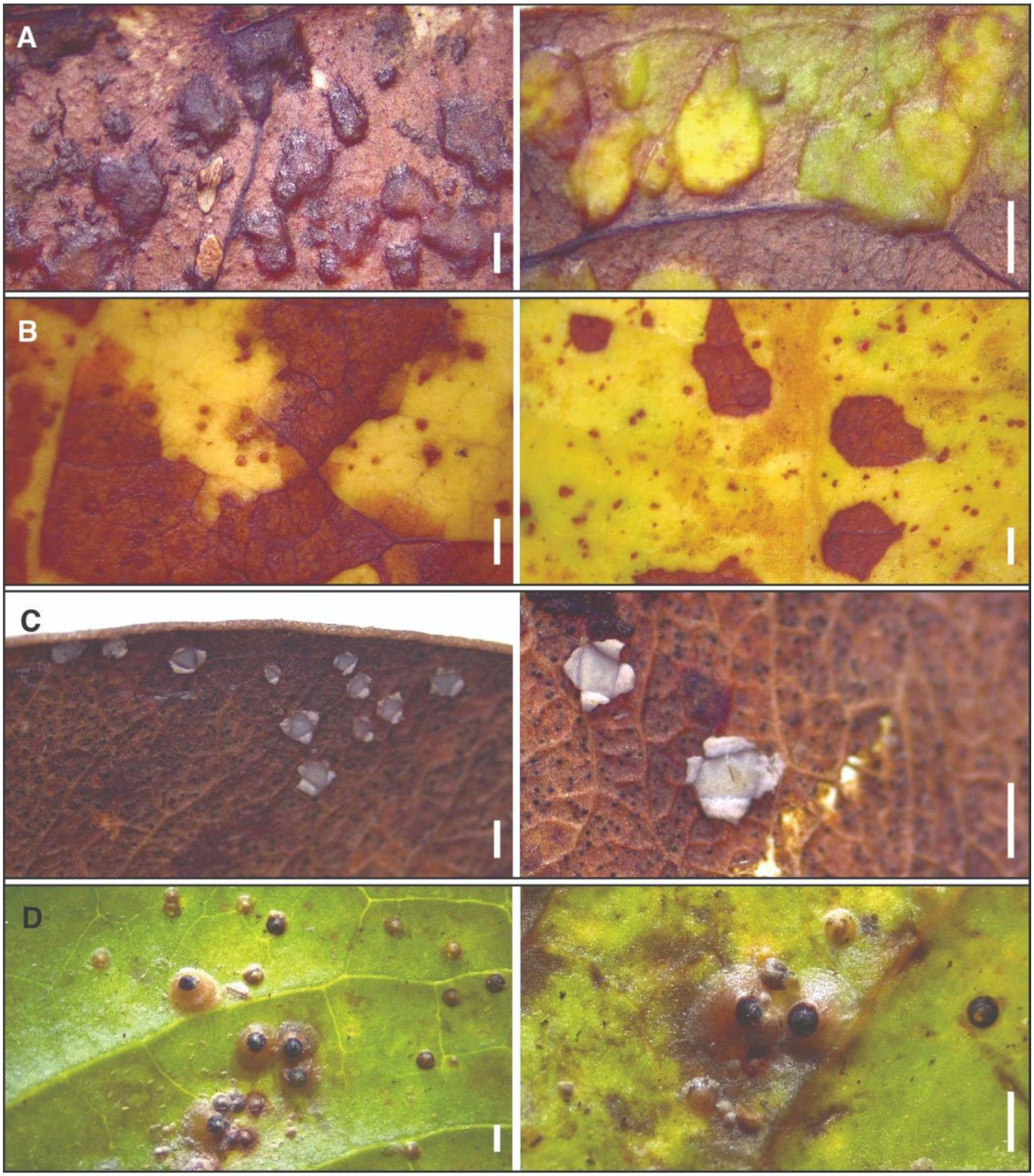

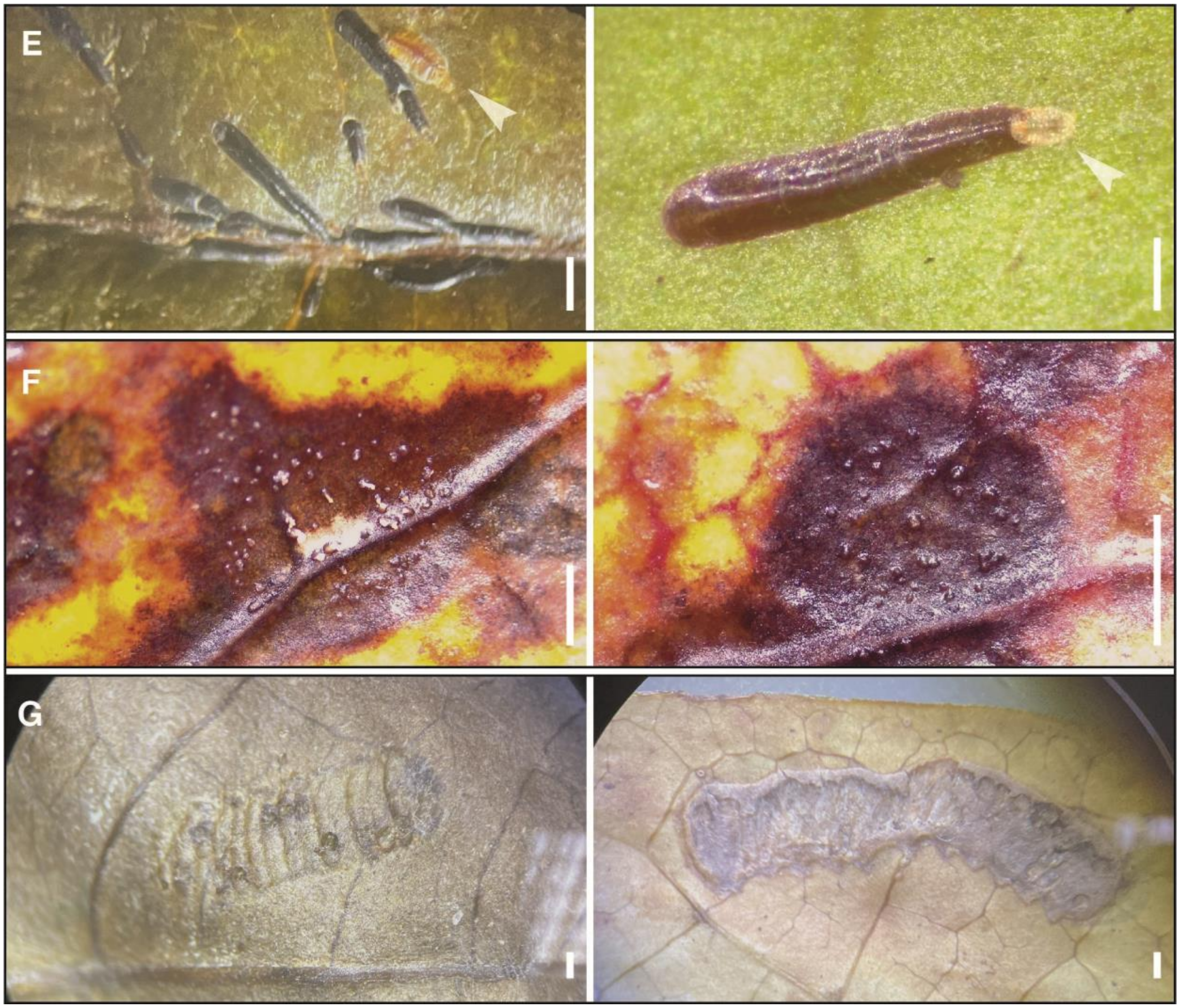

Damage types that are unique and unassignable to the previously described damage types in Labandeira et al. (2007), from the tropical lowland rainforest of the ‘Ōpūnohu Valley, Mo‘orea, French Polynesia. (A**)** MDT01; polylobate and blotched, raised fungal damage. (B**)** MDT02; irregularly shaped, darkened and slightly indented patches of fungal damage. (C**)** MDT03; circular white fungal patches (~1 mm) rimmed by triangular flaps that are raised on the adaxial leaf surface. (D**)** MDT04; shallowly conical, exophytically attached oviposition structures (< 1 mm) on the abaxial leaf surface with a centrally darkened, well-defined center. (E**)** MDT05; linear to curvilinear exophytic oviposition chamber, likely made by scale insects (white arrow). (F**)** MDT06; patches of clustered piercing & sucking damage within darkened leaf tissue. (G**)** MDT07; endophytic oviposition with 15-20 parallely laid, slightly curved and elongated ellipsoid eggs. Right image showing the post-hatch trace. The scale bar is 1 mm.
