# Supplementary material for: Invasive plants reduce functional feeding diversity and trophic interactions of insect herbivores on a remote tropical island
Source: PLoS One. 2026 Jun 11;21(6):e0349238. doi: 10.1371/journal.pone.0349238 (PMC13257969; doi:10.1371/journal.pone.0349238)
Supplement: S3 File — (PDF) [file pone.0349238.s009.pdf]

S3 File. Comparisons of damage type (DT) richness and frequency by functional feeding groups and feeding classes across native, naturalized, and invasive plants in the ʻŌpūnohu rainforest of Moʻorea, French Polynesia.

This file contains supplementary figures and summary statistics of DT richness and frequency by functional feeding groups and feeding classes across host plant categories. The DT richness by functional feeding group is presented in Fig 3 of the main text.

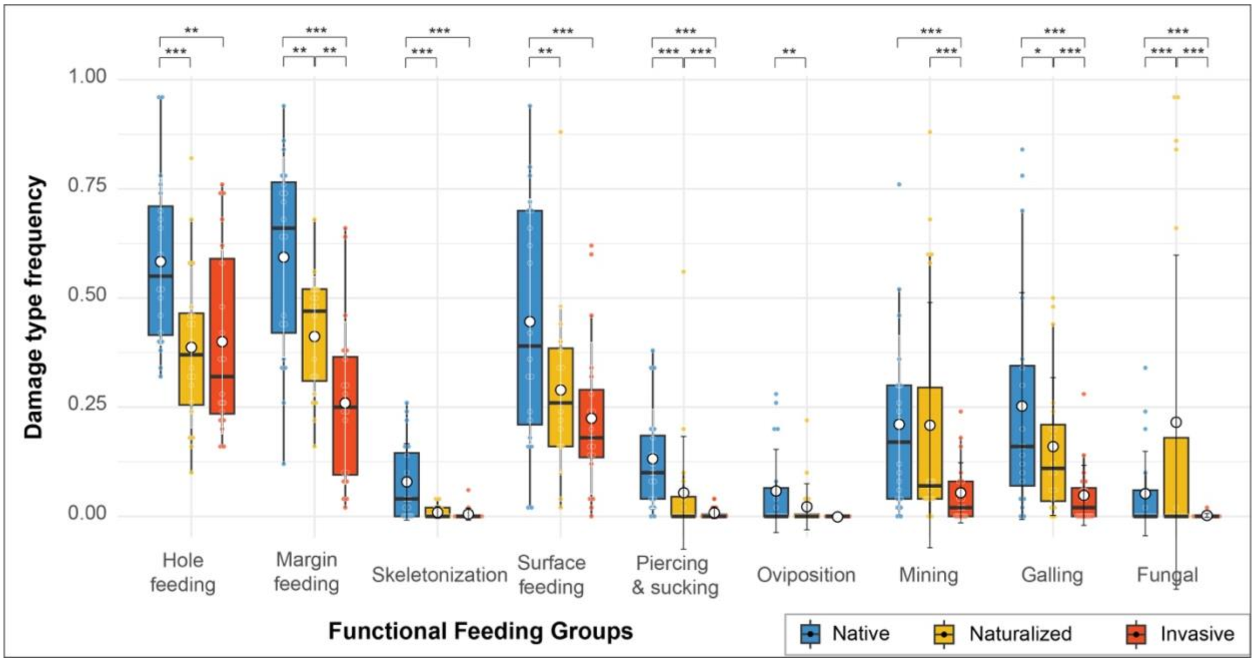

S3 File–Fig 1. Damage type frequency of different functional feeding groups across native, naturalized, and invasive plants. Boxplots show the mean (white circle), median (mid-bar), standard deviation (bracketed vertical bar), upper and lower quartiles (box), maximum and minimum values (whiskers), and individual tree values (dots). Asterisks indicate statistical significance between host plant categories (*p < 0.05, **p < 0.01, ***p < 0.001).

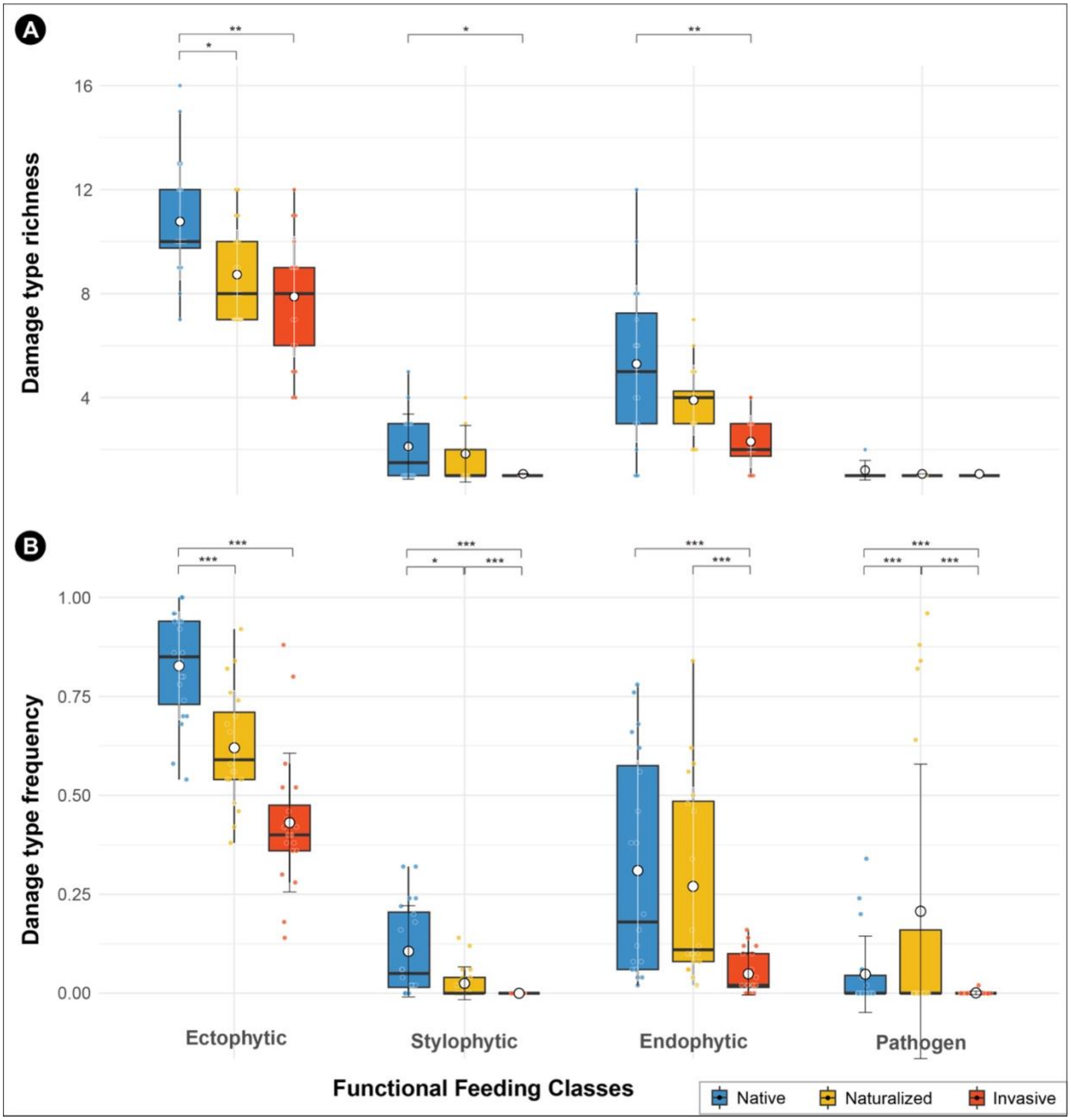

S3 File–Fig 2. Damage type richness (A) and frequency (B) by feeding classes across native, naturalized, and invasive plants. Boxplots show the mean (white circle), median (mid-bar), standard deviation (bracketed vertical bar), upper and lower quartiles (box), maximum and minimum values (whiskers), and individual tree values (dots). Asterisks indicate statistical significance between host plant categories (*p < 0.05, **p < 0.01, ***p < 0.001).

S3 File–Table 1. Statistical comparison of DT richness and frequency by functional feeding groups across host plant categories.

Abbreviations: N = native, R = naturalized, I = invasive; MF = margin feeding, HF = hole feeding, SF = surface feeding, SK = skeletonization, PS = piercing & sucking, OV=oviposition, GA=gall, MI=mining, and FU=fungal damage). Asterisks indicate statistical significance (*p < 0.05, **p < 0.01, ***p < 0.001).

	**HF**	**MF**	**SK**	**SF**	**PS**	**OV**	**MI**	**GA**	**FU**
**DT richness**									
Host plant category x Functional feeding group: F = 2.6347, p < 0.0001***									
N–R (p-adj)	0.046*	0.410	0.045*	0.956	0.032*	0.905	0.002**	0.758	0.956
N–I (p-adj)	0.064	0.010**	0.009**	0.956	0.015*	0.208	<0.001***	<0.001***	0.582
R–I (p-adj)	0.989	0.208	0.837	1.000	0.956	0.410	0.032*	0.004**	0.758
**DT Frequency**									
Host plant category x Functional feeding group: F = 25.701, p< 0.0001***									
N–R (p-adj)	<0.001***	0.002**	<0.001***	0.007**	<0.001***	0.007**	0.998	0.045*	<0.001***
N–I (p-adj)	0.002**	<0.001***	<0.001***	<0.001***	<0.001***	0.999	<0.001***	<0.001***	<0.001***
R–I (p-adj)	0.974	0.004**	0.464	0.231	<0.001***	0.999	<0.001***	<0.001***	<0.001***

S3 File–Table 2. Statistical comparisons of DT richness and frequcncy by feeding classes across host plant categories.

Abbreviations: N = native, R = naturalized, I = invasive. Asterisks indicate statistical significance (*p < 0.05, **p < 0.01, ***p < 0.001).

	**Ectophytic**	**Stylophytic**	**Endophytic**	**Pathogen**
**DT richness** Host plant category x Feeding Class: F = 1.2989, p = 0.261				
N–R (p-adj)	0.037*	0.212	0.183	0.825
N–I (p-adj)	0.003**	0.035*	0.002**	0.831
R–I (p-adj)	0.515	0.642	0.112	0.971
**DT frequency** Host plant category x Feeding class: F = 43.688, p < 0.001***				
N–R (p-adj)	<0.001***	0.042*	0.944	<0.001***
N–I (p-adj)	<0.001***	<0.001***	<0.001***	<0.001***
R–I (p-adj)	0.609	<0.001***	<0.001***	<0.001***
