# Supplementary material for: Invasive plants reduce functional feeding diversity and trophic interactions of insect herbivores on a remote tropical island
Source: PLoS One. 2026 Jun 11;21(6):e0349238. doi: 10.1371/journal.pone.0349238 (PMC13257969; doi:10.1371/journal.pone.0349238)
Supplement: S4 File — (PDF) [file pone.0349238.s010.pdf]

S4 File. Host specificity (functional breadth) of damage types across native, naturalized, and invasive study plants in the ‘Ōpūnohu lowland rainforest of Mo‘orea, French Polynesia.

Backgrounds.—Analogous to the monophagous, oligophagous, and polyphagous dietary breadths of insect herbivores, DTs exhibit an array of functional breadths (host specificity). These range from specialized (occurring on a single host plant or a few closely related species in the same family) to intermediate (on closely related plant families) and generalized (on distantly related host species across orders) [1,2]. The DTs are functional units, and a single insect species can produce multiple DTs across its life cycle, and conversely, a single DT can be made by multiple herbivore species with similar functional morphology and behavior relating to their feeding [2,3], which makes the DTs a trait-based system without one-to-one pairings between the DTs and the damage makers [4]. Therefore, DT functional breadths are analogous, but not equivalent, to taxonomic dietary breadth.

Assessment of host specificity (taxonomic dietary breadth as well as dietary breadth) depends on taxon sampling of host plants. While extensive community-level sampling of plants and herbivores/DTs provides more accurate measures of host specificity, such efforts are time- and labor-intensive and are largely qualitative, and quantitative studies typically sample a small fraction of the local flora, with taxon sampling structured according to host plant phylogeny (genus- or family-level relatedness) or ecology (growth forms, successional status) (see [5] for more). Because we limited our taxon sampling to twelve species that are abundant in the forest, our study does not capture the full potential DT host range. Our assessment of the DT functional breadths presented herein is to support our assessment of the specificity of plant–DT interactions, complementary to the bipartite network analysis.

Methods.—We scored DT functional breadths on twelve study taxa, following the classifications by Xiao et al. [2], defining specialized, intermediate, and generalized DTs based on their occurrence range across the host plants. **Specialized** DTs are defined as DTs occurring on a narrow range of confamilial host plants; **intermediate** as occurring on host plants across closely related families, and **generalized** as occurring across distantly related species across orders. Possible intermediate categorization in this study includes occurrences across Verbenaceae (*D. erecta* and *L. camara*) and Bignoniaceae (*S. campanulata*; Lamiales), or Melastomataceae (*M. calvescens*) and Myrtaceae (*S. malaccense*, *S. cumini*).

To account for herbivore errors or identification inaccuracies [5], DTs with at least three occurrences were assigned a functional breadth. Damage type functional breadths were analyzed in two metrics: DT richness (using an LMM with an interaction between functional breadth categories and host plant types) and DT frequency (using a binomial GLMM; including trees and species as random effects).

Patterns by DT functional breadths were analyzed in two metrics—DT richness and frequency across host plant categories. Damage type richness by functional breadths was

compared using an LMM with an interaction between functional breadth and host plant categories, with species as random effects. Frequency was tested using a binomial GLMM including species as random effects.

Results.—Of the 79 total identified DTs, we excluded 26 rare DTs (fewer than three total occurrences) from functional breadth analyses; these included 23 single-species occurrences and three cases of two occurrences across distantly related families. Among the 53 DTs meeting the occurrence threshold, 46 were categorized as generalized and 7 as specialized (S4 File–Table 1). Notably, all seven specialized DTs were restricted to native and naturalized plants, including piercing-and-sucking (MDT06) and mining (DT37) damages on *B. asiatica*; oviposition on *M. collina* (MDT02) and *D. erecta* (MDT04, MDT05); and fungal pathogens on *N. forsteri* (MDT02) and *M. collina* (MDT03). We did not recover DTs of intermediate functional breadth that occurred on closely related families within the same order in this study.

Both DT richness and frequency by functional breadths varied significantly across host plant categories ($p < 0.001$ for both). Regarding richness, generalized DTs were most diverse on native plants (mean 16.5), followed by naturalized (12.8) and invasive (9.7) categories, with significant differences between all pairs. Specialized DTs were present on naturalized (1.5) and native (1.2) plants, but were entirely absent on invasive species (S4 File–Fig 1A).

Invasive plants received substantially less frequent generalized DTs (67.8%) compared to native (95.0%) and naturalized (86.5%) plants. For specialized DTs, native plants recorded the highest frequency (8.1%), which was higher than naturalized (1.3%) and invasive (0%) plants. The percentage of undamaged leaf samples was highest in invasive plants (31.7%) and lowest in native species (3.4%), with an intermediate value for naturalized ones (10.7%) (S4 File–Fig 1B).

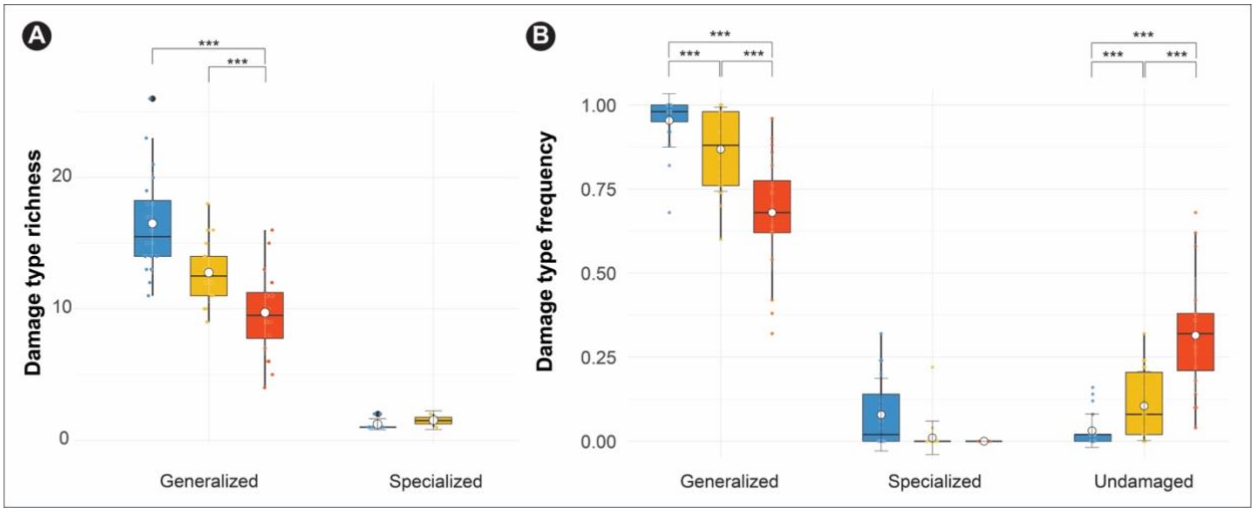

S4 File–Fig 1. Damage type richness (A) and frequency (B) by functional breadths across native, naturalized, and invasive plants in the ʻŌpūnohu rainforest. Note: no DTs of intermediate functional breadth were recovered in this study. Boxplots show the mean (white circle), median

(mid-bar), standard deviation (bracketed vertical bar), upper and lower quartiles (box), maximum and minimum values (whiskers), and individual tree values (dots). Asterisks indicate statistical significance in differences across host plant categories (* $p < 0.05$, ** $p < 0.01$, *** $p < 0.001$).

S4 File–Table 1. Assessment of the DT functional breadth, based on their occurrence on the twelve host plant taxa.

Damage type	Number of DT occurrences on leaf litter samples (N=3,000)	Number of host plants occurrence (N = 12)	DT specificity score based on the host species occurrence
**Hole feeding**			
DT01	864	12	1: Generalized
DT02	165	12	1: Generalized
DT03	292	12	1: Generalized
DT04	8	4	1: Generalized
DT05	115	12	1: Generalized
DT06	8	2	1: Generalized
DT07	1	1	N/A
DT08	4	3	1: Generalized
DT09	3	3	1: Generalized
DT51	4	4	1: Generalized
DT57	1	1	N/A
DT63	4	3	1: Generalized
DT64	5	5	1: Generalized
DT68	1	1	N/A
DT78	2	2	N/A
**Margin feeding**			
DT12	917	12	1: Generalized
DT13	267	12	1: Generalized
DT14	17	9	1: Generalized
DT15	72	10	1: Generalized
DT81	32	9	1: Generalized
**Skeletonization**			
DT16	75	8	1: Generalized
DT17	1	1	N/A
DT20	1	1	N/A
DT24	6	2	1: Generalized
DT61	8	2	1: Generalized

DT79	2	1	N/A
**Surface feeding**			
DT25	8	3	1: Generalized
DT29	817	12	1: Generalized
DT30	143	12	1: Generalized
DT31	1	1	N/A
**Piercing & Sucking**			
DT46	149	10	1: Generalized
DT47	19	3	1: Generalized
DT48	2	1	N/A
DT128	1	1	N/A
MDT06	20	1	3: Specialized
**Oviposition**			
DT54	1	1	N/A
DT67	5	2	1: Generalized
DT76	3	3	1: Generalized
DT100	1	1	N/A
DT101	45	4	1: Generalized
DT102	9	1	3: Specialized
MDT04	6	1	3: Specialized
MDT05	8	1	3: Specialized
MDT07	3	2	1: Generalized
**Galling**			
DT32	398	12	1: Generalized
DT33	2	2	N/A
DT34	7	5	1: Generalized
DT52	27	3	1: Generalized
DT62	1	1	N/A
DT84	3	2	1: Generalized
DT85	4	2	1: Generalized
DT116	9	3	1: Generalized
DT119	3	2	1: Generalized
DT122	1	1	N/A

DT125	1	1	N/A
DT127	1	1	N/A
DT144	4	2	1: Generalized
DT145	6	4	1: Generalized
DT146	1	1	N/A
DT147	2	2	N/A
**Mining**			
DT35	65	12	1: Generalized
DT36	187	12	1: Generalized
DT37	3	1	3: Specialized
DT41	49	4	1: Generalized
DT42	1	1	N/A
DT43	2	1	N/A
DT45	109	3	1: Generalized
DT59	1	1	N/A
DT66	16	7	1: Generalized
DT69	60	8	1: Generalized
DT90	5	3	1: Generalized
DT104	2	1	N/A
DT109	15	3	1: Generalized
DT131	1	1	N/A
**Fungal damage**			
DT58	1	1	N/A
MDT01	216	3	1: Generalized
MDT02	41	1	3: Specialized
MDT03	8	1	3: Specialized
**Incertae sedis**			
DT106	1	1	N/A
**Undamaged**			
Undamaged	461	-	-

S4 File–Table 2. Statistical summary of DT richness and frequency by functional breadth categories across native, naturalized, and invasive plants. DT richness was analyzed using an LMM, and DT frequency was compared using a binomial GLMM. Asterisks indicate statistical significance in differences across host plant categories (*p < 0.05, **p < 0.01, ***p < 0.001).

	Generalized	Specialized	Undamaged
**<u>DT richness</u>**			
Host plant category x Functional breadths: F = 4.6603, p = 0.035*			
N–R	0.0142*	0.7025	
N–I	<0.001***	N/A	
R–I	0.0215*	N/A	
**<u>DT frequency</u>**			
Host plant category x Functional breadths: F = 129.820, p < 0.001***			
N–R	<0.001***	0.992	<0.001***
N–I	<0.001***	N/A	<0.001***
R–I	<0.001***	N/A	<0.001***

References

1. Labandeira CC, Wilf P, Johnson K, Marsh F. Guide to Insect (and Other) Damage Types on Compressed Plant Fossils. Washington, D. C.: Smithsonian Institution; 2007 pp. 1–25. Available: https://www.researchgate.net/publication/271076149

2. Xiao L, Chen L, Labandeira CC, Azevedo-Schmidt L, Wang Y, Ren D. The modern pattern of insect herbivory predates the advent of angiosperms by 60 My. Proc Natl Acad Sci USA. 2025;122: e2412036122. doi:10.1073/pnas.2412036122

3. Carvalho MR, Wilf P, Barrios H, Windsor DM, Currano ED, Labandeira CC, et al. Insect leaf-chewing damage tracks herbivore richness in modern and ancient forests. López-Vaamonde C, editor. PLoS ONE. 2014;9: e94950. doi:10.1371/journal.pone.0094950

4. Swain A, Maccracken SA, Fagan WF, Labandeira CC. Understanding the ecology of host plant–insect herbivore interactions in the fossil record through bipartite networks. Paleobiology. 2022;48: 239–260. doi:10.1017/pab.2021.20

5. Novotny V, Basset Y. Host specificity of insect herbivores in tropical forests. Proc R Soc B. 2005;272: 1083–1090. doi:10.1098/rspb.2004.3023
